# Impact of *Lactobacillus casei* BL23 on the Host Transcriptome, Growth and Disease Resistance in Larval Zebrafish

**DOI:** 10.3389/fphys.2018.01245

**Published:** 2018-09-04

**Authors:** Chubin Qin, Yadong Xie, Yibing Wang, Shuning Li, Chao Ran, Suxu He, Zhigang Zhou

**Affiliations:** Key Laboratory for Feed Biotechnology of the Ministry of Agriculture, Feed Research Institute, Chinese Academy of Agricultural Sciences, Beijing, China

**Keywords:** probiotics, mRNA sequencing, development, immunomodulation, disease resistance

## Abstract

In this study, zebrafish were treated with *Lactobacillus* strains as probiotics from hatching to puberty, and the effect of treatment with *L. casei* BL23 on the development and immunity response of the host was investigated. Genes that were differentially expressed (DEGs) in the overall body and intestine were detected at 14 days post fertilization (dpf) and 35 dpf, respectively, using whole transcriptome sequencing (mRNAseq). We showed that zebrafish raised by continuous immersion with *L. casei* BL23 showed a higher final body weight at 14 dpf (*P* < 0.05), and 35 dpf (*P* < 0.01). DEGs between *L. casei* BL23 treatment and control group at 14 dpf were involved in myogenesis, cell adhesion, transcription regulation and DNA-binding and activator. At 35 dpf, 369 genes were DEGs in the intestine after treatment with *L. casei* BL23, which were involved in such categories as signaling, secretion, motor proteins, oxidoreductase and iron, tight junctions, lipid metabolism, growth regulation, proteases, and humoral and cellular effectors. KEGG analysis showed DEGs to be involved in such pathways as those associated with tight junctions and the PPAR signal pathway. RT-*q*PCR analysis showed that expression of insulin-like growth factors-I (*igf1*), peroxisome proliferator activated receptors-α (*ppar-α*) and -β (*ppar-*β), Vitamin D receptor-α (*vdr-α*), and retinoic acid receptor-γ (*rar-γ*) was up-regulated in fish treated with *L. casei* BL23 at 35 dpf. After 35 days of treatment, the mortality rate in *L. casei* BL23 treated group was lower than the control after challenge with *A. hydrophila* (*P* < 0.05), and the pro-inflammatory cytokine *il-1*β, anti-inflammatory cytokine *il-10* and complement component 3a (*c3a*) showed more expression in *L. casei* BL23 group at 8h after challenge, 24 h after challenge, or both.. Together, these data suggest that specific *Lactobacillus* probiotic strains can accelerate the development profile and enhance immunity in zebrafish, which supports the rationale of early administration of probiotics in aquaculture.

## Introduction

Probiotics are live microorganisms that can confer health benefits on the host when delivered in adequate amounts (FAO/WHO, 2001). The beneficial effects of probiotics involved in fish nutrition, dysbiosis counteraction, gut homeostasis and health, growth promotion, immune enhancement and other effects have been well documented (Aureli et al., 2011; Zorriehzahra et al., 2016). In addition, probiotics have been widely used in the aquaculture industry for disease control and growth promotion (Michael et al., 2014).

Zebrafish have many characteristics that favor their use in host-microbe interactions research. These include the available of wealth genetic resources, the transparency during early developmental stages that permits real-time observation of host and microbial cells *in vivo*, and short life cycle (Lieschke and Currie, 2007). These features, combined with extensive homologies between the zebrafish and mammalian genomes, made the zebrafish a useful model for basic and biological research (Grunwald and Eisen, 2002). In addition, the zebrafish has been proven to be a practical model for studying the probiotic effects of *Lactobacillus*, such as lowering lipid levels (Falcinelli et al., 2015), attenuating high-fat diet-related metabolic disorders (Falcinelli et al., 2017) and anxiety-related behavior (Davis et al., 2016), promoting growth (Avella et al., 2012), and promoting disease resistance (Wang et al., 2015; He et al., 2017).

After the fry hatch, the gastrointestinal tract (GIT) of zebrafish is colonized by microbes from the living environment within 12–24 h, concurrent with the differentiation of GIT (Bates et al., 2006; Rawls et al., 2007). At 4 days post fertilization (dpf), within a day after the opening of the mouth, the populations of colonized bacteria increase because of swallowing (Bates et al., 2006). In particular, early gut colonization of microbes plays important roles in the morphological and immunological development of the GIT, and development of a functional fermentative environment and neonatal pathogen resistance (Yeoman and White, 2014). Previous studies showed that probiotic administration during early developmental stages of fish was more effective than during other stages and that it could increase survival (Gatesoupe, 2008) and growth of larvae (Avella et al., 2010, 2012). Gut adhesion (colonization) is recognized as important parameter of a potential probiotics (Havenaar et al., 1992; Salminen et al., 1998). However, our preliminary work showed that both the highly adhesive gut strain *L. rhamnosus* JCM 20300 (Zhou et al., 2012) and the non-adhesive strain *L. casei* BL23 (Qin et al., 2014) can accelerate zebrafish growth. This might indicate that the gut colonization seems not to be a very important criterion for a probiotic effect. In order to acquire more basic knowledge regarding the non/less gut adhesive strain *L. casei* BL23 and its effects on the developmental profile and immunity education in zebrafish, which favor the potential use of the probiotic in aquaculture, the whole-transcriptome sequencing (mRNA-seq) was performed at 14 dpf (whole body) and 35 dpf (intestine), respectively, following exposure to the *Lactobacillus*.

## Materials and Methods

### Bacteria and Culture Condition

*Lactobacillus rhamnosus* GG, *L. rhamnosus* JCM 20300, *L. plantarum* JCM 1149, *Lactobacillus casei* BL23 and *L. acidophilus* JCM 1132 were from our laboratory stock, and were cultivated in MRS (De Man, Rogosa, Sharpe) broth at 37°C for 24 h. *Aeromonas hydrophila* NJ-1 was grown in Luria–Bertani (LB) broth for 18 h at 37°C, 200 rpm. *Lactobacilli* and *A. hydrophila* NJ-1 cells were collected by centrifugation (10 min, 4000 × g, 4°C). The pellets then washed with sterile water three times, and resuspended in sterile water to a final concentration of 1.0 × 10^10^ CFU /ml.

### Animals

Adult zebrafish (*Danio rerio*) (TU line) were obtained from the Center of Developmental Biology and Genetics, College of Life Sciences, Peking University, China. The fish were kept in tanks in a recirculation aquaculture system at 28 ± 0.5°C, with a 14 h light, 10 h dark photoperiod. The inlet water flow was approximately 1 L/min. The fish were fed to visual satiation two times a day with freshly hatched brine shrimp (8:30 a.m. and 5:30 p.m.). The protocol was approved by the animal ethics committee of Feed Research Institute, Chinese Academy of Agricultural Sciences (2017-ZZG-ZF-002).

### Administration of Potential Probiotics and Husbandry Conditions

Four pairs of adult zebrafish (TU strain) were mated and used to produce embryos. The embryos were collected and mixed together, and then the eggs were washed three times with sterile water. The embryos were randomly divided into six experimental tanks, with 150 embryos in each tank. During the hatching period, the dead embryos were removed and an equal number of healthy embryos were added. After hatching, the larvae were kept in tanks in a static water system at 28 ± 0.5°C, with a 14-h light, 10-h dark photoperiod. From 3 dpf, the suspensions of *L. rhamnosus* GG, *L. rhamnosus* 20300, *L. plantarum* JCM 1149, *Lactobacillus casei* BL23, and *L. acidophilus* JCM 1132 were added to the water containing the zebrafish larvae at a final concentration of 10^6^ CFU/ml, respectively. The frequency of probiotics administration was every 48 h after water renewal to maintain survival of the probiotics in the water (**Supplementary Figure S1**). In the control group, the same volume of water was added. Larvae were fed *Paramecium* at 5 dpf at early stages (5–14 dpf), and were fed brine shrimp nauplii from 15 dpf onward. The larvae and juveniles were sacrificed and weighed at 14 and 35 dpf for analysis of growth performance. This experiment was used to select the best probiotic that promotes zebrafish growth and use the probiotic strain for further analysis at later study.

### *Lactobacillus casei* BL23 Administration

The first experiment showed that both *L. casei* BL23 and *L. rhamnosus* 20300 can accelerate the growth of zebrafish. In addition, our previous study found that *L. casei* BL23 administration could stimulate follicle maturation, enhance fecundity and improve egg quality in zebrafish (Qin et al., 2014), and we were very interested in why this non/less adhesive stain was able to confer these beneficial effects on the host. Accordingly, we used *L. casei* BL23 for further analysis in zebrafish growth performance and immunity education. After hatching, the larvae were administrated live or dead (98°C, 20 min) cells of *L. casei* BL23 at a final concentration of 10^6^ CFU/ml in the water. These live and dead cells were added every 48 h after water renewal. In the control group, the same volume of water was added. Larvae were fed with equal amount of *Paramecium* from 5 dpf to 14 dpf, and brine shrimp nauplii from 15 dpf onward. The amount of feed was increased by 3–5% every 5 days.

The larvae and juveniles were sacrificed and weighed at the indicated points in time for sample collection. At 14 dpf, 30 larvae were randomly selected from each tank (*n* = 4) from different treatment groups sacrificed, and weighed, then collected in the frozen RNase-free tubes at -20°C directly upon sampling and stored at -80°C. At 35 dpf, 15 juveniles (were randomly) selected from each tank (*n* = 4) from different treatment groups sacrificed, and weighed, then dissected on ice. The intestines were collected and stored at -80°C.

### Intestinal Retention of *Lactobacillus*

After 35 days of BL23 administration, the fish was fasted for 12 h and three fish from each tank from each treatment were sacrificed (*n* = 12) and their guts were sampled. Each gut was homogenized with 1 mL PBS, respectively, and the homogenate was serially diluted with PBS. Specific dilutions were cultured on MRS plates at 37°C for 48 h. The CFUs on the MRS plates were calculated as *Lactobacillus* counts.

### Challenge Test and Cytokine Expression Analysis

After 35 days of BL23 administration, the fish (40 fish per tank) were challenged with *A. hydrophila* NJ-1 at 5 × 10^8^ CFU/mL. Mortality was recorded for 26 days. At 0, 8, 24, and 48 h post-infection, three fish of each tank (*n* = 4) from each treatment were randomly collected and sacrificed. The intestinal tissues of each tank were collected and pooled and then immediately frozen in liquid nitrogen and stored at -80°C for cytokine expression analysis. The total RNA was isolated from the pooled intestine samples with TRIzol (Tian Gen, Beijing, China) in accordance with the manufacturer’s specifications. The protocol of RT-qPCR is described below.

### mRNAseq: Total RNA Isolation

Total RNA was isolated from pooled samples of larvae whole body or juvenile’s intestines with TRIzol (Tian Gen, Beijing, China)in accordance with the manufacturer’s specifications. The RNA was treated with amplification-grade DNase I (1 U/μg RNA; Invitrogen, United States). RNA concentration was measured using Qubit^®^ RNA Assay Kit in Qubit^®^ 2.0 Fluorometer (Life Technologies, CA, United States). RNA integrity was assessed using the RNA Nano 6000 Assay Kit of the Bioanalyzer 2100 system (Agilent Technologies, CA, United States) (Liu et al., 2015). All the RNA RIN values were bigger than 9.5 as analyzed by Bioanalyzer 2100 system. For mRNAseq, equal volume and amount (1 μg) of total RNA from 4 sample pools of each treatment group were mixed as a sequencing sample. However, for qPCR analysis, four replicated sample pools from each treatment group were used independently.

### mRNA-Seq: Library Preparation and Sequencing

For RNA-seq, 1 μg of total RNA of each sample was used for library preparation, and the NEBNext^®^ Ultra^TM^ RNA Library Prep Kit for Illumina^®^ (NEB, United States) was used in accordance with the manufacturer’s specifications and index codes were added to distinguish sequences from each sample. Libraries concentration was quantitated by qPCR with Illumina Library Quantification kit (KAPA BioSystems, Cape Town, South Africa). Sequencing was performed by Novogene Bioinformatics Technology Co., Ltd., Beijing, China on an Illumina Hiseq 2000 platform and 100 bp single-end reads were generated. For each library, approximately 25 to 30 million reads were obtained.

### mRNAseq: Data Analysis

Raw data were first processed through in-house Perl scripts. Clean reads were obtained by removing reads containing adapter or reads containing ploy-*N*, and low-quality reads from raw reads. Single-end clean reads were aligned to the reference genome (ZV9 zebrafish genome) using TopHat v2.0.9 with two mismatches. HTSeq v0.6.1 was used to count the reads numbers mapped to each gene. Differential expression analysis of two conditions was performed using the DEGSeq R package (1.12.0). The *P*-values were adjusted using the Benjamini and Hochberg method. Corrected *P*-value of 0.05 and log2 (fold change) of one were set as the threshold for significantly differential expression. The differentially expressed genes (DEGs) were functionally classified and characterized by using DAVID 6.8. The raw data are available from BioProject PRJNA428924.

### Real-Time PCR

The *q*PCR protocol is described in our previous work (Qin et al., 2014). In brief, 1 μg of total RNA was used for *c*DNA synthesis with a TransScript One-Step *g*DNA Removal and *c*DNA Synthesis SuperMix (TransGen, China) in a 20-μL volume. Two microliter portions of diluted (1/10) cDNA were used to perform *q*PCR by SYBR Green Supermix (TianGen, China) in an CFX iCycler thermal cycler (Bio-Rad) in a 20-μL volume, with final concentrations of forward and reverse primers as 0.5 μM. The reaction mixtures were incubated for 5 min at 95°C, followed by 40 cycles of 20 s at 95°C, 20 s at 60°C and 20 s at 72°C, and finally the melt curve was graphed from 65°C to 95°C with a 0.5°C increment for 10 s. Housekeeping genes of rpl13 and rps11 were used as references. For each *c*DNA sample, three wells were used for *q*PCR amplification as the technical replicates. 2^-ΔΔC_t_^ was used to quantify and normalize gene expression. The sequences of specific primers are presented in **Table 1**.

**Table 1:** List of primers used for real-time PCR analyses.

Genes	Sequence (5′to 3′)
c3aF	ATGAGCTCCTGCAGAGGTGT
c3aR	AGTGGTTGTTGGAGGTCTGG
IGF-1aF	GGCAAATCTCCACGATCTCTAC
IGF-1aR	CAGTTCATTCCTCCCGCTGT
PPAR-α F	TCCACATGAACAAAGCCAAA
PPAR-α R	AGCGTACTGGCAGAAAAGGA
PPAR-β F	CAGGTGACGCTGCTGAAATA
PPAR-β R	CGGAGGAACTCTCTCGTCAC
VDR-α F	CTCCAGTGAGGAGGATCAGC
VDR-α R	TCTTCAGCCGTCAGGTCTCT
RAR-γ F	ATTCCGCCAGAGAGCTATGA
RAR-γ R	TAGGCCCAGGTCTAGCTGAA
IL-1β F	GAGACAGACGGTGCTGTTTA
IL-1β R	GTAAGACGGCACTGAATCCA
rps11 F	ACAGAAATGCCCCTTCACTG
rps11 R	GCCTCTTCTCAAAACGGTTG
rpl13 F	TCTGGAGGACTGTAAGAGGTATGC
rpl13 R	TCAGACGCACAATCTTGAGAGCAG
TNF-α F	CAGAGTTGTATCCACCTGTTA
TNF-α R	TTCACGCTCCATAAGACCCA
IL-10 F	ATTTGTGGAGGGCTTTCCTT
IL-10 R	AGAGCTGTTGGCAGAATGGT

### Statistical Analysis

The data of growth phenotypes, *q*PCR and mortality data are presented as mean ± SEM. For growth phenotypes data, one-way analysis of variance (ANOVA) was performed after Bonferroni’s test with GraphPad Prism version 5.0 software. In addition, for *q*PCR and mortality data analysis, unpaired *t*-test was performed. Wherever applicable, *P*-values are reported, and a *P*-value of ≤ 0.05 is considered significant.

## Results

### Growth Promotion Effect of Different *Lactobacillus* Strains Used as Probiotics

**FIGURE 1:**
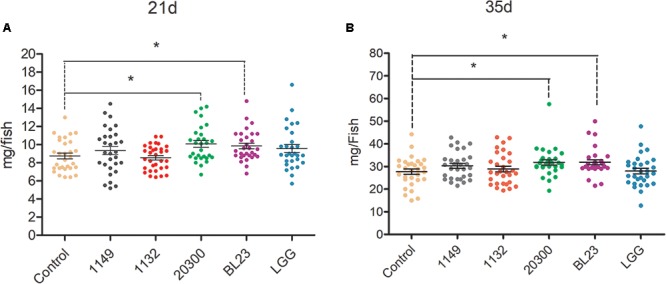
Effects of potential probiotics on growth promotion in larval zebrafish. The body weight (mg/individual) of zebrafish larvae at 21 **(A)** dpf and 35 **(B)** dpf were showed following treated with different potential probiotics. The data are shown as mean ± SEM (*n* = 35). Data were analyzed using ANOVA test followed by Bonferroni analysis. *P*-value of ≤0.05 is considered significant difference. One asterisk indicate *P* < 0.05.

Larvae treated with *L. casei* BL23 and *L. rhamnosus* 20300 showed a higher body weight at 21 dpf and 35 dpf than the control (**Figure 1**, *P* < 0.05). However, no significant difference was detected in the body weight of zebrafish receiving *L. rhamnosus* LGG, *L. plantarum* JCM 1149, or *L. acidophilus* JCM 1132 relative to control at both 21 dpf and 35 dpf (**Figure 1**).

### Retention of *Lactobacillus* in Zebrafish Juveniles

**FIGURE 2:**
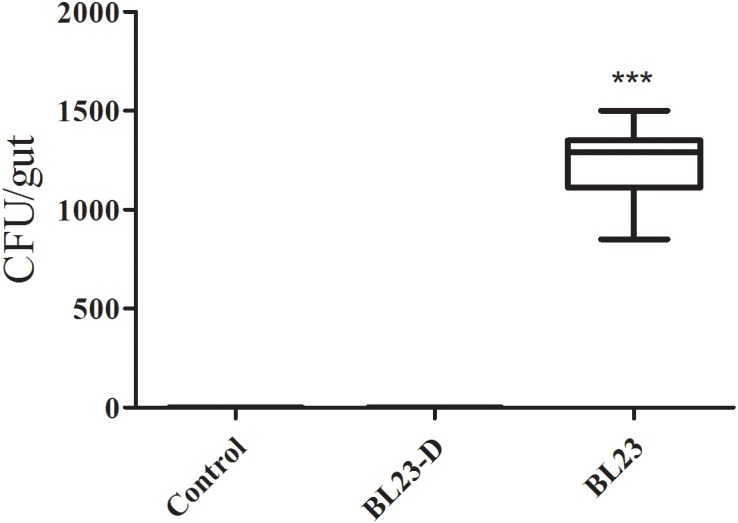
Retention of *L. casei* BL23 in the intestine of zebrafish juveniles. The data are shown as mean ± SEM (*n* = 8). Data were analyzed using ANOVA test followed by Bonferroni analysis. *P*-value of ≤0.05 is considered significant difference. Trible asterisk indicate *P* < 0.001.

No *Lactobacillus* was detected in the intestines of fish in the control or dead *L. casei* treatment groups (**Figure 2**). However, in the live *L. casei* treatment group, the amount of *L. casei* BL23 recovered from the intestine of zebrafish was 1.24 × 10^3^ CFU/fish (**Figure 2**).

### Viability of Bacterial Cells and Growth Promotion of *L. casei* BL23

**FIGURE 3:**
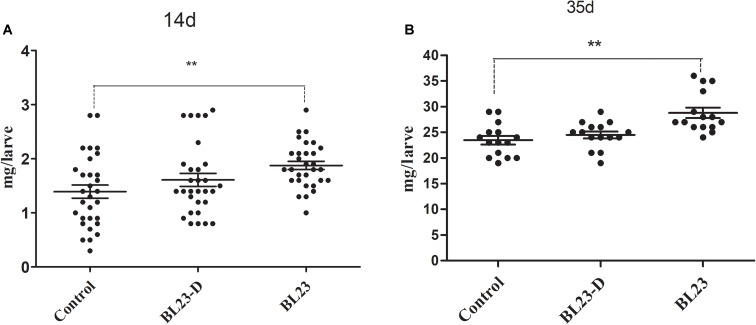
Growth promotion of *L. casei* BL23 depends on the viability the bacteria cells. The body weight (mg/individual) of zebrafish larvae at 21 **(A)** dpf and 35 **(B)** dpf were showed following treated with live or dead cells of *L. casei* BL23, respectively. The data are shown as mean ± SEM (*n* = 30 at 14 dpf and *n* = 15 at 35 dpf, respectively). Data were analyzed using ANOVA test followed by Bonferroni analysis. *P*-value of ≤0.05 is considered significant difference. One asterisk indicate *P* < 0.05. Double asterisk indicates *P* < 0.01.

To further study the growth promotion effect of *L. casei* BL23 in zebrafish, we tested the effect of both live and dead *L. casei* BL23 cells in larval zebrafish. We found that the growth promoting effect of *L. casei* BL23 was dependent on its viability (**Figure 3**). Larvae treated with live *L. casei* BL23 showed a higher body weight at 14 dpf and 35 dpf compared with the control (**Figure 3**, *P* < 0.05). However, no significant difference from control was observed in the body weight of zebrafish treated with dead cells of *L. casei* BL23 (**Figure 3**).

### mRNAseq Gene Expression Analyses

To gain insight into the mechanisms underlying the growth promotive effect of *L. casei* BL23, we performed a high-throughput gene expression analysis using mRNA-seq to compare the transcriptomics between control and BL23-treated zebrafish at 14 dpf (whole body) and 35 dpf (intestine), respectively. The mapping data were shown in **Table 2**. The results of heat map and PCA analysis of gene expression pattern are shown in **Supplementary Figures S2**, **S3**. A different pattern of genes expression was observed between control and BL23 treated fish at 35 dpf (**Supplementary Figures S2**, **S3**).

**Table 2:** The total mapping reads and percentage to genome of each sample analyzed.

Sample name	Control-14d	BL23-14d	Control-35d	BL23-35d
Total reads	25161888	29347458	31755534	27986406
Total mapped	21582221 (85.77%)	25183290 (85.81%)	26767878 (84.29%)	23954875 (85.59%)
Multiple mapped	909053 (3.61%)	944937 (3.22%)	735319 (2.32%)	671108 (2.4%)
Uniquely mapped	20673168 (82.16%)	24238353 (82.59%)	26032559 (81.98%)	23283767 (83.2%)
Non-splice reads	10400282 (41.33%)	11945903 (40.71%)	13404075 (42.21%)	11403612 (40.75%)
Splice reads	10272886 (40.83%)	12292450 (41.89%)	12628484 (39.77%)	11880155 (42.45%)

**FIGURE 4:**
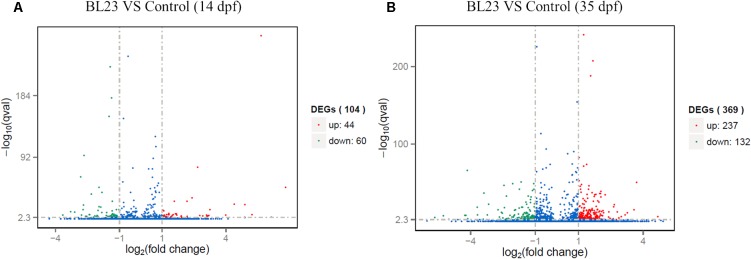
Differentially expressed genes of whole body of zebrafish at 14 dpf **(A)** and intestine of zebrafish 35 dpf **(B)** respectively, following treated with *L. casei* BL23 vs. control. Log2 (Fold change) ≥1 were set as the threshold for significantly differential expression.

At 14 dpf, a total of 104 genes were differentially expressed after treatment with *L. casei* BL23 (**Figure 4A** and **Supplementary Table S1**). Among them, 44 genes were up-regulated (log2 (fold change) > 1) and 60 genes were down-regulated (log2 (fold change) < -1) compared with control (**Figure. 4A**). DAVID analysis revealed that the DEGs were involved in myogenesis, cell adhesion, transcription regulation, and DNA-binding and activation (**Supplementary Table S2**). Genes related to myogenesis, the regulation of transcription, and DNA-binding and activation were mostly up-regulated (**Supplementary Table S2**), whereas genes involved in cell adhesion were all down-regulated (**Supplementary Table S2**).

**FIGURE 5:**
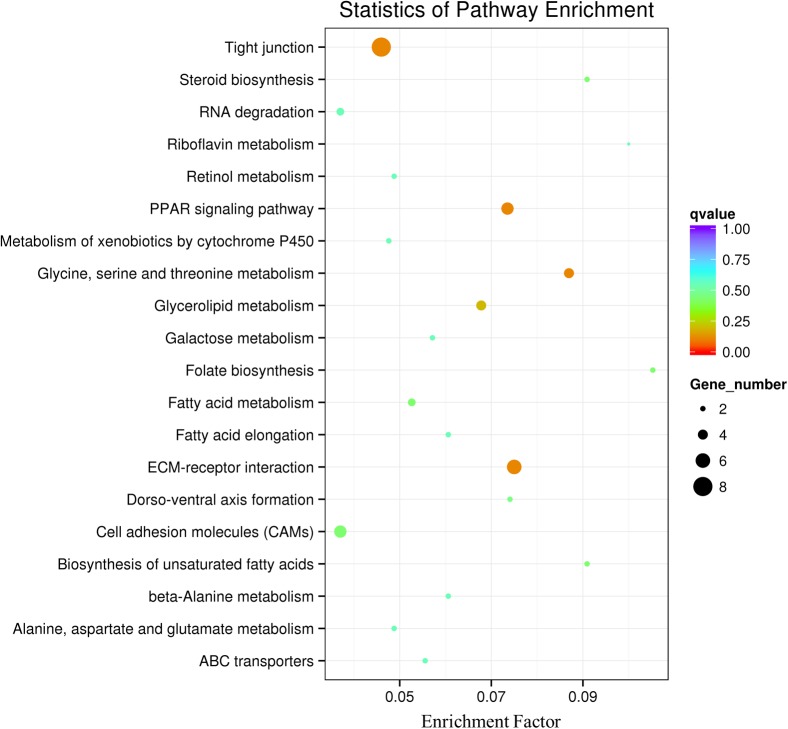
Pathway classification based on KEGG enrichment analysis of differentially transcribed genes in the intestine of zebrafish following BL23 treatment. Genes with mRNA showing at least 2-fold change are shown; adjusted *p* ≤ 0.05 for all data selected.

At 35 dpf, a total of 369 genes were differentially expressed in the BL23 group vs. control (**Figure 4B** and **Supplementary Table S3**). Of these, 237 genes were up-regulated and 132 genes were down-regulated (**Figure 4B**). To facilitate the presentation and interpretation of results, DEGs were functionally categorized. The DEGs are involved in signaling, secretion, motor protein, oxidoreductase, and iron, tight junctions, lipid metabolism, growth regulation, protease and humoral, and cellular effectors, and their levels of expression were up-regulated in the most genes except those associated with motor proteins (**Supplementary Tables S4**, **S5**). In addition, KEGG analysis showed that DEGs much enrich involved tight junction, ECM-receptor interaction, and the PPAR signal pathway (**Figure 5**).

### RT-qPCR Analysis

**FIGURE 6:**
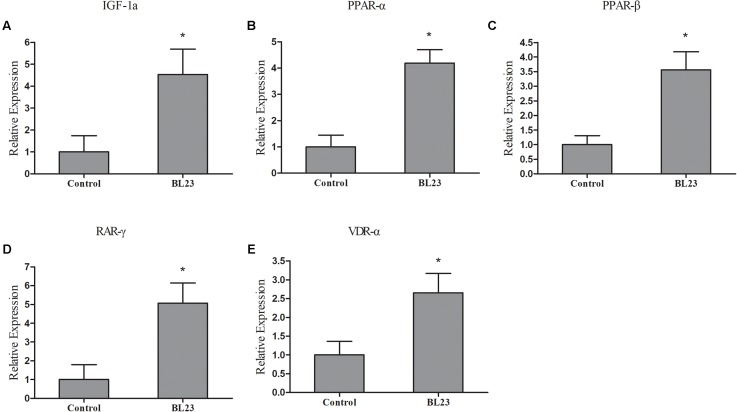
The mRNA levels of *igf1a* **(A)**, *ppar-α* **(B)**, *ppar-*β **(C)**, *rar-γ* **(D)**, and *vdr-α* **(E)** in the intestine of zebrafish treated with *L. casei* BL23 and control at 35 dpf. The independent triplicates of total RNA extraction and corresponding cDNA synthesis were carried out with the treated fish from different tanks (*n* = 4) and the results are given as mean ± SEM. Asterisk denotes a significant difference (*P* < 0.05) as analyzed using ANOVA test followed by Bonferroni analysis.

RNA-seq analysis showed that *L. casei* BL23 could regulate the expression of genes involved in myogenesis, the regulation of growth, and the PPAR signal pathway in zebrafish (**Supplementary Table S6**). Here, the representative genes involved in growth promotion and metabolism were analyzed using RT-*q*PCR (**Figure 6**). At 35 dph, the levels of expression of the genes *igf-1*, *ppar-α*, *ppar-*β, *rar-γ*, and *vdr-α* were significantly higher in the *L. casei* BL23 administration group than among controls (**Figure 6**, *P* < 0.05).

### *L. casei* BL23 and Infection of Zebrafish by *A. hydrophila*

**FIGURE 7:**
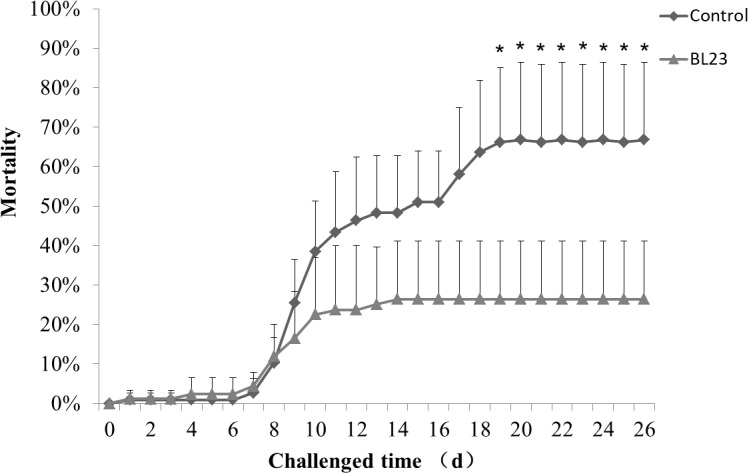
Accumulated mortality rates (%) of zebrafish treated with *L. casei* BL23 and control when challenged with *A. hydrophila* NJ-1 starting at 35 dpf for 26 days. Each data represents the mean of four replicate tanks (*n* = 28/tank), a single asterisk representation of *P*-value < 0.05.

After 35 days of BL23 administration, fish were challenged with *A. hydrophila* NJ-1. The cumulative mortality of fish treated with *L. casei* BL23 was significantly lower than the control, indicating that BL23 has a protective effect in zebrafish upon *A. hydrophila* infection (**Figure 7**, *P* < 0.05).

### Immuno-Regulatory Effect of *L. casei* BL23

**FIGURE 8:**
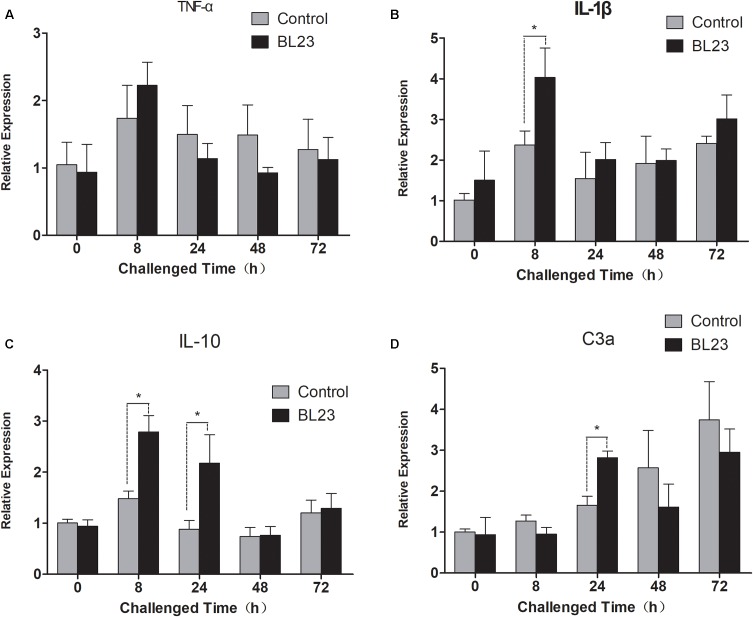
The mRNA levels of *tnf-α* **(A)**, *il-1*β **(B)**, *il-10* **(C)**, and *c3a* **(D)** in the intestine of zebrafish treated with *L. casei* BL23 and control at the indicated time when challenged with *A. hydrophila* NJ-1. The independent quadruplicates of total RNA extraction and corresponding cDNA synthesis were carried out with the treated fish from different tanks (*n* = 4) and the results are given as mean ± SEM. Asterisk denotes a significant difference (*P* < 0.05) as analyzed using ANOVA test followed by Bonferroni analysis.

After challenge with *A. hydrophila* NJ-1, the intestinal expression of cytokine genes was assessed at different points in time. The levels of expression of tumor necrosis factor -α (*tnf-α*) showed no significant difference between the BL23 group and control after 0, 8, 24, 48, and 72 h of infection (**Figure 8A**). However, a higher level of interleukin-1β (*il-1*β) expression was detected in BL23 group than in the control group at 8 h post-infection (**Figure 8B**, *P* < 0.05). Similarly, more expression of interleukin-10 (*il-10*) was observed in the BL23 group at 8 and 24 h than in the control group (**Figure 8C**, *P* < 0.05). In addition, the level of expression of complement component c3a (*c3a*) was higher in BL23 group than in the control group at 24 h after infection (**Figure 8D**, *P* < 0.05).

## Discussion

Probiotics are bacteria that provide health benefits to the host. However, the mechanism by which these bacteria promote growth and modulate the immune response conferred by a potential probiotic strain is not well understood yet. In this study, zebrafish larvae were given one of the highly adhesive strains of *L. rhamnosus* LGG, *L. plantarum* JCM 1149 and *L. rhamnosus* JCM 20300 or the non/less adhesive strains of *L. casei* BL23 and *L. acidophilus* JCM 1132 from birth to puberty. We found that zebrafish treated with *L. casei* BL23 or *L. rhamnosus* 20300 showed improved growth. This indicated that the effects of probiotics do not depend on the adhesive properties of the gut but rather on some trait inherent in the strains. We showed that the growth promotive effect of *L. casei* BL23 relies on the viability of BL23 cells. This indicated that the metabolites of *L. casei* BL23, the production of which requires the bacterial cells to remain viable, might play important roles in zebrafish growth.

Beneficial microbes such as LAB may increase the height of the villi in the host and thereby increase surface absorptive area in the intestine, which contributes to more efficient use of diet-derived energy sources (Pirarat et al., 2011). Moreover, LAB can produce some metabolic substrates, such as vitamins (LeBlanc et al., 2011), short chain fatty acids, organic acids, and digestive enzymes (Bairagi et al., 2002; John et al., 2006). These are involved in host nutrient metabolism and development. In the current study, we used mRNA-seq to investigate the molecular differentiation of physiological processes in larval zebrafish as indicated by the different expression genes. At 14 dpf, 104 genes were found to be differentially expressed. Functional classification of genes showed most to be involved in cell adhesion, the regulation of transcription, myogenesis, and DNA-binding, and most of them were upregulated in the BL23 group. Genes of *hsp90a* and *unc45b* were upregulated in the *L. casei* BL23 group. These are known to mediate the folding, assembly and accumulation of thick-filament myosin during the formation of sarcomeres, and to play a critical role in the development of striated muscle and the stability of sarcomere (Hawkins et al., 2008; Hu et al., 2016). Integrin alpha (6B) has been shown to be involved in chondrogenic cell differentiation (Segat et al., 2002). These data indicate that *L. casei* BL23 mainly influenced the progress of cell growth and differentiation during the early development of zebrafish.

At 35 dpf, intestinal mRNAseq showed that 369 genes were differentially expressed after treatment with *L. casei* BL23; these were involved in categories such as signaling, oxidoreductase, and iron, tight junction, lipid metabolism, growth regulation, protease, and humoral effectors, cellular effectors and others. KEGG analysis showed DEGs to be involved in such pathways as tight junction, PPAR signal pathway, and glycine, serine, and threonine metabolism. Fish growth is a complex process, which is positively correlated with muscle growth and is mainly regulated by the GH/IGF system (Reinecke et al., 2005; Wood et al., 2005). GH is a pituitary hormone that regulates numerous physiological processes, including immune function, somatic growth, protein, and lipid metabolism, and feeding behavior (Kawauchi and Sower, 2006). As expected, we found that growth regulation genes *igf1*, *igf2*, *igfbp2a*, *igfbp2b*, and *igfals* to be upregulated in zebrafish treated with *L. casei* BL23 at 35 dpf. A higher level of transcription of *igf1* mRNA were observed by RT-qPCR in the *L. casei* BL23 treatment group. Avella’s work showed that administration of *L. rhamnosus* IMC 501 accelerated growth in the false percula clownfish (Avella et al., 2010) and zebrafish (Avella et al., 2012), and the mRNA levels of the of factors involved in growth and development such as *igf1*, *ppar-α*, *ppar-*β, *vdr-α*, and *rar-γ* were higher in the probiotic treatment group (Avella et al., 2010, 2012).

Short fatty acids and vitamins are the natural products of *Lactobacillus* fermentation. They are known to activate PPARs, which are involved in skeletal development, lipid metabolism, and cell proliferation (Krey et al., 1997; Grimaldi, 2007; Neschen et al., 2007). Vitamin D and retinoic acid are essential molecules for organism morphogenesis and chondrogenesis (Rhinn and Dolle, 2012; Eyles et al., 2013). In this study, as expected, the nuclear receptors *vdrα*, *rarγ*, and *ppar*s were found to be up-regulated in BL23 group as indicated by DGE and RT-qPCR analysis. These findings suggested that positive correlation between these systems and the growth accelerated involved in metabolites of BL23.

Immunomodulation is one of the most common benefits associated with probiotics. The immunity modulation role of probiotics in aquatic animals has been reported extensively (Nayak, 2010; Perez et al., 2010; Dimitroglou et al., 2011; Lazado and Caipang, 2014; Lazado et al., 2015). The protective effects that probiotics confer on their hosts usually involve competitive exclusion of pathogens (Vine et al., 2004; Chabrillon et al., 2005), production of antibacterial compounds such as antibiotics and bacteriocins (Gibson, 1999; Birri et al., 2013), or reinforcement of the gut epithelial barrier in the gut (Candela et al., 2008; Mennigen and Bruewer, 2009). In addition, probiotics can interact with intestinal epithelial cells via pattern recognition receptors (such as Toll-like receptors), which can modulate physiological and immunological responses in hosts, such as cytokine production, antigen presentation, and regulatory T cell differentiation and regulation of function, and these processes are crucial to host defense from disease (Kawai and Akira, 2010; Lebeer et al., 2010). Previous works have indicated that probiotics (such as lactic acid bacteria) can effectively regulate the expression of pro-inflammatory cytokines such as IL-1, IL-6, IL-12, TNF-α, gamma interferon (IFN-γ), and anti-inflammatory cytokines such as IL-10 and TGF-β in animals (Christensen et al., 2002; Niers et al., 2005; Ashraf et al., 2014). In this study, genes of the tight junction complex (*cldn15a*, *cldnc*, *cldnd*, and *cldng*) and humoral effectors (c5, *c3b.1*, and *c3b.2*) were significantly up-regulated in the intestines of zebrafish treated with *L. casei* BL23. The mortality of zebrafish is a result of a long-term *A. hydrophila* infection and hunger, as observed in our lab. Zebrafish treated with BL23 showed a lower mortality rate upon challenge with pathogen *A. hydrophila*. The gene expression levels of pro-inflammatory cytokine *il-1*β, anti-inflammatory cytokine *il-10* and complement component *c3a* were higher in the BL23 treatment group at 8 h or 24 h after challenge with *A. hydrophila*. Galindo and his co-workers (Galindo-Villegas et al., 2012) showed that colonization by commensal microbes in newly hatched zebrafish primes neutrophils and induces several genes encoding pro-inflammatory and antiviral mediators via TLR/MyD88 signaling pathway, which increased the resistance of larva to viral infection. For this reason, we here speculate that BL23 may increase the resistance of zebrafish to infection by regulating host intestinal barrier function and modulating cytokine expression.

In summary, our results demonstrated that continuous administration of probiotic strain *L. casei* BL23 during zebrafish development promoted growth performance and increased disease resistance in zebrafish. The results obtained in this study indicate the potential use of the probiotic strain for fish growth promotion and diseases controls in aquaculture.

## Author Contributions

All authors made substantial contributions to the design of the work, acquisition and analysis of data for the work, and drafting the work.

## Conflict of Interest Statement

The authors declare that the research was conducted in the absence of any commercial or financial relationships that could be construed as a potential conflict of interest.

## Supplementary Material

FIGURE S1 The alive of probiotic bacterial cells in the water at different time point after administration.

FIGURE S2 Cluster analysis of differentially expressed genes.

FIGURE S3 PCA analysis of differentially expressed genes.

TABLE S1 Difference of genes expression between control group and treatment group zebrafish at 14 dpf.

TABLE S2 Functional gene groups and their differentially expressed genes in larvae zebrafish at 14 dpf treated with *L. casei* BL23 vs. control.

TABLE S3 Difference of genes expression between control group and treatment group zebrafish at 35 dpf.

TABLE S4 Functional gene groups and their differentially expressed genes in larvae zebrafish at 35 dpf treated with *L. casei* BL23 vs. control.

TABLE S5 Functional gene groups and their differentially expressed genes in larvae zebrafish at 35 dpf treated with *L. casei* BL23 vs. control.

TABLE S6 Genes involved myogenesis, growth regulation and PPAR signal pathway in zebrafish at 35 dpf, *L. casei* BL23 vs. control.
