# Supplementary figures and images for: Impact of Lactobacillus casei BL23 on the Host Transcriptome, Growth and Disease Resistance in Larval Zebrafish
Source: Front Physiol. 2018 Sep 4;9:1245. doi: 10.3389/fphys.2018.01245 (PMC6131626; doi:10.3389/fphys.2018.01245)

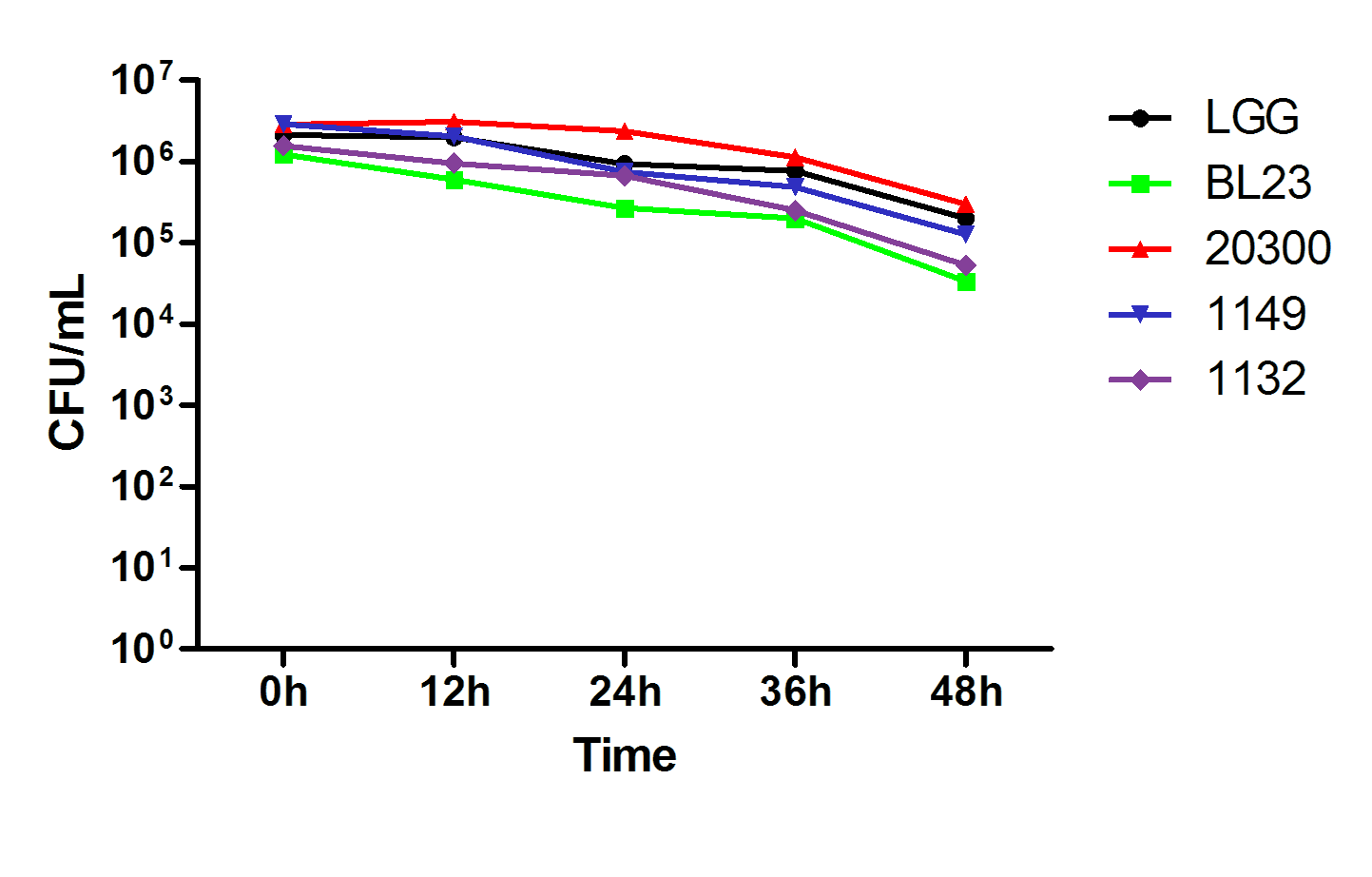

Supplement: FIGURE S1 — The alive of probiotic bacterial cells in the water at different time point after administration. [file Image_1.TIF]

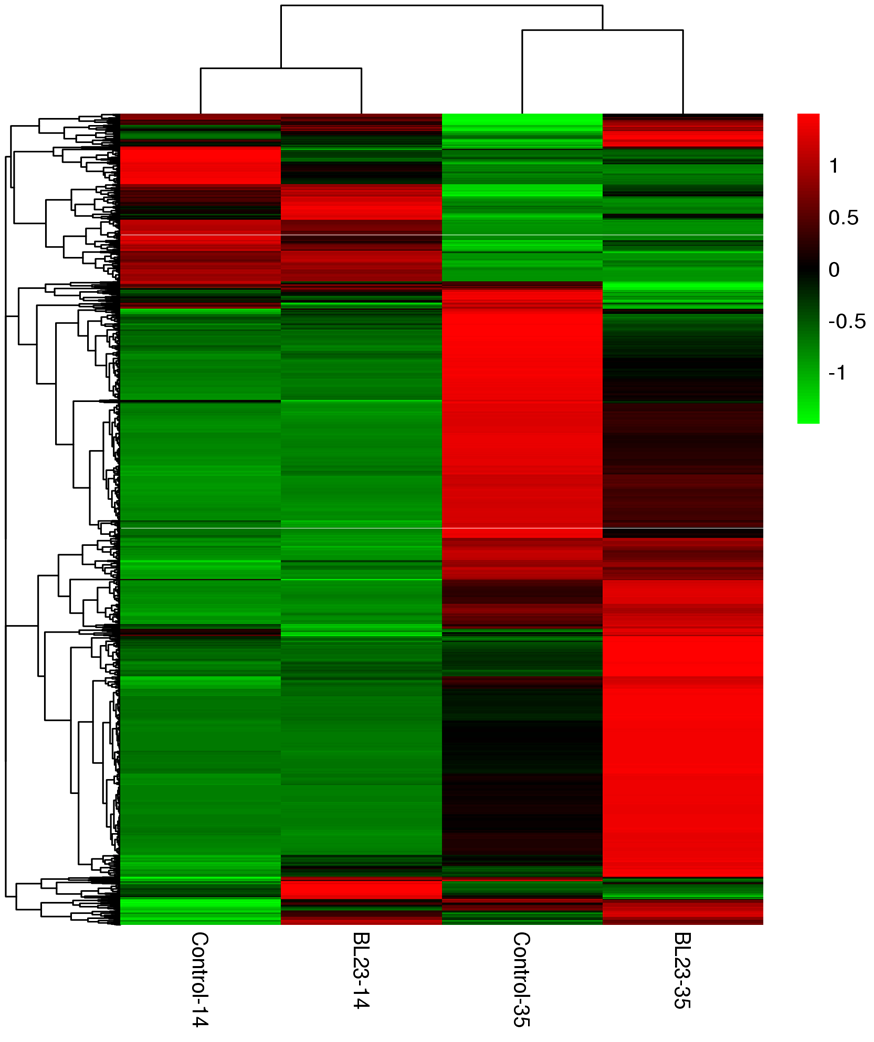

Supplement: FIGURE S2 — Cluster analysis of differentially expressed genes. [file Image_2.TIF]

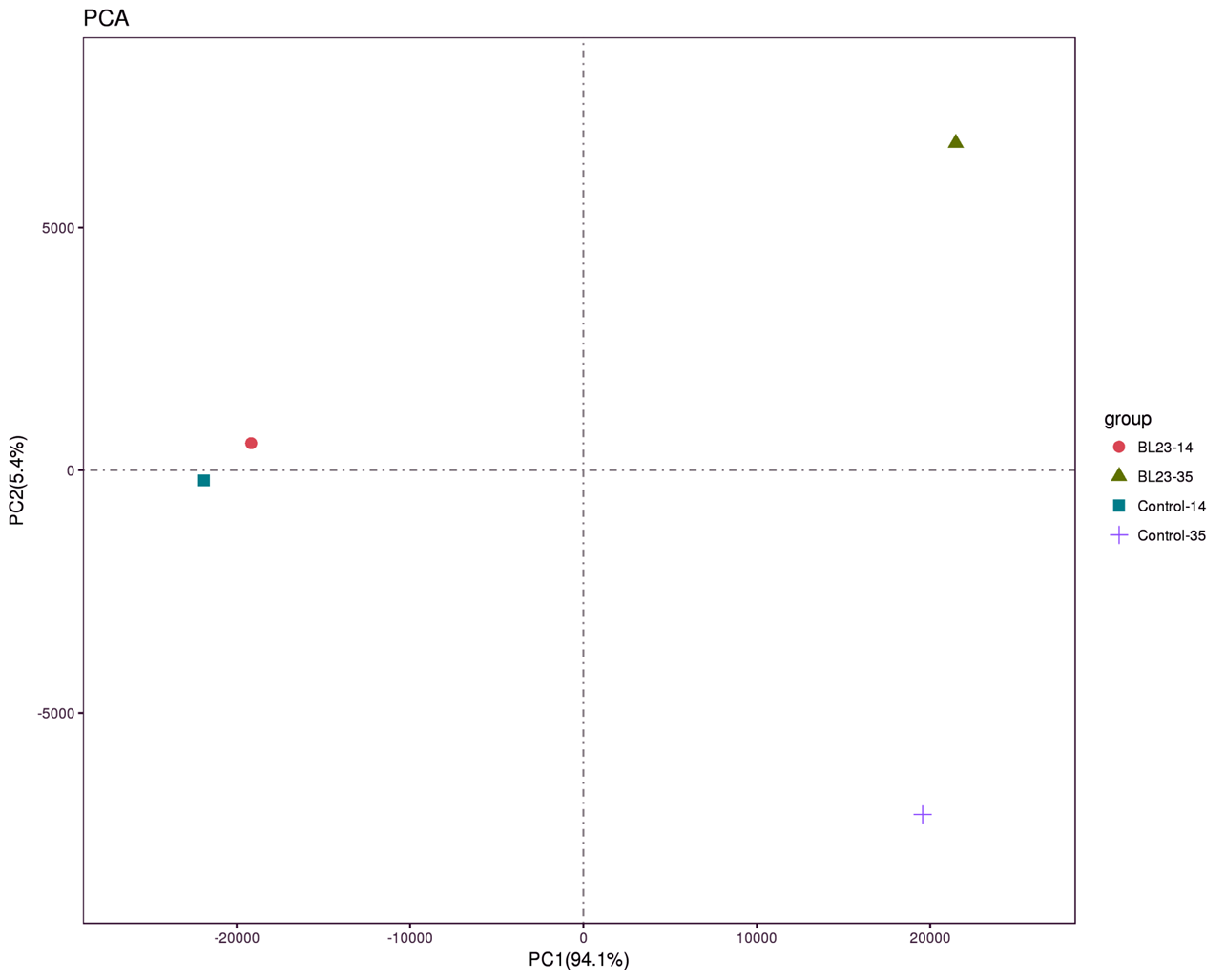

Supplement: FIGURE S3 — PCA analysis of differentially expressed genes. [file Image_3.TIF]
