# Supplementary material for: Impact of Lactobacillus casei BL23 on the Host Transcriptome, Growth and Disease Resistance in Larval Zebrafish
Source: Front Physiol. 2018 Sep 4;9:1245. doi: 10.3389/fphys.2018.01245 (PMC6131626; doi:10.3389/fphys.2018.01245)
Supplement: TABLE S2 — Functional gene groups and their differentially expressed genes in larvae zebrafish at 14 dpf treated with L. casei BL23 vs. control. [file Table_2.DOCX]

Table S2 Functional gene groups and their differentially expressed genes in larvae zebrafish at 14 dpf treated with *L. casei* BL23 vs control

| ENSEMBL_GENE_ID | Gene Name | log2.Fold_change. | p-value |
| --- | --- | --- | --- |
| Myogenesis | | | |
| ENSDARG00000010478 | heat shock protein 90, alpha (cytosolic), class A member 1, tandem duplicate 1(hsp90aa1.1) | 1.5758 | 4.05E-30 |
| ENSDARG00000008433 | unc-45 myosin chaperone B(unc45b) | 1.1035 | 9.33E-06 |
| Cell adhesion | | | |
| ENSDARG00000039677 | desmocollin 2 like(dsc2l) | -2.3231 | 1.08E-40 |
| ENSDARG00000069946 | integrin, alpha 6b(itga6b) | -2.8164 | 1.21E-66 |
| ENSDARG00000028507 | integrin, beta 4(itgb4) | -2.6613 | 6.18E-99 |
| Transcription regulation and DNA-binding | | | |
| ENSDARG00000020133 | Jun dimerization protein 2b(jdp2b) | 3.1528 | 4.42E-07 |
| ENSDARG00000037421 | early growth response 1(egr1) | 1.6765 | 1.09E-10 |
| ENSDARG00000044010 | lysyl oxidase-like 2a(loxl2a) | -2.5373 | 6.35E-07 |
| ENSDARG00000055752 | neuronal PAS domain protein 4a(npas4a) | 3.0936 | 1.47E-08 |
| ENSDARG00000030616 | nuclear factor, erythroid 2-like 1a(nfe2l1a) | -1.3693 | 5.04E-06 |
| ENSDARG00000007241 | v-myc avian myelocytomatosis viral oncogene homolog b(mycb) | 1.449 | 6.68E-06 |
| ENSDARG00000055751 | FBJ murine osteosarcoma viral oncogene homolog B(fosb) | 4.3934 | 3.81E-25 |
| ENSDARG00000031683 | v-fos FBJ murine osteosarcoma viral oncogene homolog Ab(fosab) | 3.2466 | 5.39E-17 |
| Activator | | | |
| ENSDARG00000037421 | early growth response 1(egr1) | 1.6765 | 1.09E-10 |
| ENSDARG00000055752 | neuronal PAS domain protein 4a(npas4a) | 3.0936 | 1.47E-08 |
| ENSDARG00000007241 | v-myc avian myelocytomatosis viral oncogene homolog b(mycb) | 1.449 | 6.68E-06 |
