# Supplementary material for: Impact of Lactobacillus casei BL23 on the Host Transcriptome, Growth and Disease Resistance in Larval Zebrafish
Source: Front Physiol. 2018 Sep 4;9:1245. doi: 10.3389/fphys.2018.01245 (PMC6131626; doi:10.3389/fphys.2018.01245)
Supplement: TABLE S4 — Functional gene groups and their differentially expressed genes in larvae zebrafish at 35 dpf treated with L. casei BL23 vs. control. [file Table_4.DOCX]

Table S4 Functional gene groups and their differentially expressed genes in larvae zebrafish at 35 dpf treated with *L. casei* BL23 vs control

| ENSEMBL_GENE_ID | GeneName | log2.Fold_change. | | pvalue | |
| --- | --- | --- | --- | --- | --- |
| Secreted | | | | |  |
| ENSDARG00000075159 | antigen p97 (melanoma associated) identified by monoclonal antibodies 133.2 and 96.5(mfi2) | 1.422 | 6.97E-33 | |  |
| ENSDARG00000012076 | apolipoprotein A-Ia(apoa1a) | -1.1431 | 0 | |  |
| ENSDARG00000029493 | coagulation factor IXb(f9b) | 1.3345 | 9.41E-05 | |  |
| ENSDARG00000079752 | collagen, type VI, alpha 4a(col6a4a) | 1.7037 | 5.74E-06 | |  |
| ENSDARG00000021004 | complement component 5(c5) | 1.2162 | 1.74E-32 | |  |
| ENSDARG00000093068 | complement component c3b, tandem duplicate 1(c3b.1) | 1.4833 | 5.03E-49 | |  |
| ENSDARG00000001818 | complement component c3b, tandem duplicate 2(c3b.2) | 1.2874 | 2.13E-30 | |  |
| ENSDARG00000012609 | hemopexin(hpx) | 1.6718 | 4.42E-211 | |  |
| ENSDARG00000057498 | hyaluronan binding protein 2(habp2) | 1.1067 | 1.52E-14 | |  |
| ENSDARG00000052470 | insulin-like growth factor binding protein 2a(igfbp2a) | 1.1657 | 1.20E-09 | |  |
| ENSDARG00000031422 | insulin-like growth factor binding protein 2b(igfbp2b) | 1.4607 | 8.00E-07 | |  |
| ENSDARG00000053476 | lipase, hepatic a(lipca) | 1.1685 | 5.02E-09 | |  |
| ENSDARG00000087697 | lipoprotein lipase(lpl) | 1.1256 | 0.00010028 | |  |
| ENSDARG00000079727 | selenoprotein P, plasma, 1b(sepp1b) | 1.0273 | 1.73E-08 | |  |
| ENSDARG00000089966 | si:zfos-1505d6.3(si:zfos-1505d6.3) | -1.6125 | 2.98E-34 | |  |
| ENSDARG00000037191 | transthyretin (prealbumin, amyloidosis type I)(ttr) | 1.0052 | 2.16E-07 | |  |
| ENSDARG00000040815 | tsukushi small leucine rich proteoglycan homolog (Xenopus laevis)(tsku) | 1.3009 | 1.03E-06 | |  |
| ENSDARG00000008948 | zgc:123275(zgc:123275) | -2.3401 | 1.74E-05 | |  |
| ENSDARG00000086352 | zona pellucida glycoprotein 2, tandem duplicate 1(zp2.1) | -1.7205 | 3.22E-22 | |  |
| ENSDARG00000090237 | zona pellucida glycoprotein 2, tandem duplicate 3(zp2.3) | -2.1825 | 2.03E-70 | |  |
| ENSDARG00000086522 | zona pellucida glycoprotein 2, tandem duplicate 5(zp2.5) | -1.7684 | 4.31E-30 | |  |
| ENSDARG00000091409 | zona pellucida glycoprotein 2, tandem duplicate 6(zp2.6) | -1.8023 | 1.31E-50 | |  |
| Motor protein | | | | |  |
| ENSDARG00000054978 | kinesin family member C3(kifc3) | -1.4035 | 3.72E-25 | |  |
| ENSDARG00000000103 | myosin, heavy chain 10, non-muscle(myh10) | 1.1409 | 2.55E-06 | |  |
| ENSDARG00000095930 | myosin, heavy chain a(myha) | -1.4959 | 5.81E-07 | |  |
| ENSDARG00000067990 | myosin, heavy polypeptide 1.1, skeletal muscle(myhz1.1) | -1.3368 | 7.91E-21 | |  |
| ENSDARG00000067995 | myosin, heavy polypeptide 1.2, skeletal muscle(myhz1.2) | -1.0152 | 5.29E-05 | |  |
| ENSDARG00000012944 | myosin, heavy polypeptide 2, fast muscle specific(myhz2) | -1.5032 | 1.74E-13 | |  |
| ENSDARG00000010332 | zgc:56231(zgc:56231) | 1.4802 | 5.02E-06 | |  |
| Oxidoreductase and iron | | | | |  |
| ENSDARG00000014646 | amine oxidase, copper containing 2(aoc2) | 1.0691 | 5.90E-23 | |  |
| ENSDARG00000068290 | cytochrome P450 CYP2X12(cyp2x12) | -1.3474 | 5.61E-07 | |  |
| ENSDARG00000021172 | cytochrome P450, family 2, subfamily AD, polypeptide 2(cyp2ad2) | 1.1941 | 6.76E-26 | |  |
| ENSDARG00000042953 | cytochrome P450, family 2, subfamily N, polypeptide 13(cyp2n13) | 1.0473 | 1.64E-07 | |  |
| ENSDARG00000045015 | cytochrome P450, family 27, subfamily B, polypeptide 1(cyp27b1) | 1.9093 | 5.43E-08 | |  |
| ENSDARG00000089369 | ferredoxin reductase(fdxr) | -1.3264 | 9.30E-09 | |  |
| ENSDARG00000043342 | glutathione peroxidase 3(gpx3) | 1.5048 | 4.39E-18 | |  |
| ENSDARG00000036942 | glycerol-3-phosphate dehydrogenase 1c(gpd1c) | -1.2678 | 4.67E-18 | |  |
| ENSDARG00000053215 | malic enzyme 1, NADP(+)-dependent, cytosolic(me1) | 1.0633 | 4.63E-12 | |  |
| ENSDARG00000019521 | myeloid-specific peroxidase(mpx) | -4.1626 | 1.18E-69 | |  |
| ENSDARG00000071429 | tryptophan 2,3-dioxygenase a(tdo2a) | 1.1461 | 5.74E-14 | |  |
| ENSDARG00000056481 | vesicle amine transport 1(vat1) | 1.42 | 9.19E-08 | |  |
| ENSDARG00000075159 | antigen p97 (melanoma associated) identified by monoclonal antibodies 133.2 and 96.5(mfi2) | 1.422 | 6.97E-33 | |  |
| ENSDARG00000004952 | radical S-adenosyl methionine domain containing 2(rsad2) | 1.7269 | 8.97E-12 | |  |
| ENSDARG00000071429 | tryptophan 2,3-dioxygenase a(tdo2a) | 1.1461 | 5.74E-14 | |  |
| Tight junction | | | | |  |
| ENSDARG00000036463 | claudin 15a(cldn15a) | 1.0397 | 2.42E-05 | |  |
| ENSDARG00000015955 | claudin c(cldnc) | 1.0282 | 7.17E-10 | |  |
| ENSDARG00000006580 | claudin d(cldnd) | 1.433 | 4.56E-11 | |  |
| ENSDARG00000003701 | claudin g(cldng) | 1.5606 | 1.42E-08 | |  |
| Lipid metabolism | | | | |  |
| ENSDARG00000004979 | ELOVL fatty acid elongase 5(elovl5) | 1.292 | 1.09E-08 | |  |
| ENSDARG00000004402 | ELOVL fatty acid elongase 6(elovl6) | 1.1996 | 1.70E-07 | |  |
| ENSDARG00000012076 | apolipoprotein A-Ia(apoa1a) | -1.1431 | 0 | |  |
| ENSDARG00000018846 | diacylglycerol O-acyltransferase 2(dgat2) | -1.0767 | 3.55E-52 | |  |
| ENSDARG00000087697 | lipoprotein lipase(lpl) | 1.1256 | 0.00010028 | |  |
| Growth regulation | | | | |  |
| ENSDARG00000052470 | insulin-like growth factor binding protein 2a(igfbp2a) | 1.1657 | 1.20E-09 | |  |
| ENSDARG00000031422 | insulin-like growth factor binding protein 2b(igfbp2b) | 1.4607 | 8.00E-07 | |  |
| ENSDARG00000037836 | insulin-like growth factor binding protein, acid labile subunit(igfals) | 1.1832 | 5.73E-06 | |  |
| Protease | | | | |  |
| ENSDARG00000010146 | carboxypeptidase A2 (pancreatic)(cpa2) | 1.2342 | 1.01E-12 | |  |
| ENSDARG00000007836 | cathepsin La(ctsla) | -1.4381 | 3.57E-28 | |  |
| ENSDARG00000074656 | cathepsin S, ortholog2, tandem duplicate 1(ctss2.1) | 1.2946 | 1.98E-07 | |  |
| ENSDARG00000036041 | coagulation factor II (thrombin)(f2) | 1.0844 | 8.27E-49 | |  |
| ENSDARG00000029493 | coagulation factor IXb(f9b) | 1.3345 | 9.41E-05 | |  |
| ENSDARG00000068181 | dipeptidase 1 (renal)(dpep1) | -1.0817 | 8.21E-09 | |  |
| ENSDARG00000057498 | hyaluronan binding protein 2(habp2) | 1.1067 | 1.52E-14 | |  |
| ENSDARG00000007988 | mannan-binding lectin serine peptidase 2(masp2) | 1.0222 | 1.71E-05 | |  |
| ENSDARG00000017676 | matrix metallopeptidase 2(mmp2) | 1.1365 | 2.16E-05 | |  |
| Humoral and cellular effectors | | | | |  |
| ENSDARG00000021004 | complement component 5(c5) | 1.2162 | 1.74E-32 | |  |
| ENSDARG00000093068 | complement component c3b, tandem duplicate 1(c3b.1) | 1.4833 | 5.03E-49 | |  |
| ENSDARG00000001818 | complement component c3b, tandem duplicate 2(c3b.2) | 1.2874 | 2.13E-30 | |  |
