# Supplementary material for: Impact of Lactobacillus casei BL23 on the Host Transcriptome, Growth and Disease Resistance in Larval Zebrafish
Source: Front Physiol. 2018 Sep 4;9:1245. doi: 10.3389/fphys.2018.01245 (PMC6131626; doi:10.3389/fphys.2018.01245)
Supplement: TABLE S5 — Functional gene groups and their differentially expressed genes in larvae zebrafish at 35 dpf treated with L. casei BL23 vs. control. [file Table_5.DOCX]

Table S5 Functional gene groups and their differentially expressed genes in larvae zebrafish at 35 dpf treated with *L. casei* BL23 vs control

| ENSEMBL_GENE_ID | GeneName | log2.Fold_change. | pvalue |
| --- | --- | --- | --- |
| Signal | | | |
| ENSDARG00000056462 | C-reactive protein 2(crp2) | 1.3203 | 5.54E-05 |
| ENSDARG00000053620 | Epstein-Barr virus induced 3(ebi3) | 1.3968 | 4.43E-05 |
| ENSDARG00000006372 | UDP glucuronosyltransferase 5 family, polypeptide C2(ugt5c2) | 1.8183 | 4.01E-05 |
| ENSDARG00000019763 | acid phosphatase 5a, tartrate resistant(acp5a) | 1.5927 | 7.30E-07 |
| ENSDARG00000053774 | alkaline phosphatase, intestinal, tandem duplicate 2(alpi.2) | -1.2861 | 2.78E-11 |
| ENSDARG00000056314 | alpha-2-macroglobulin-like(a2ml) | 1.3807 | 4.55E-77 |
| ENSDARG00000014646 | amine oxidase, copper containing 2(aoc2) | 1.0691 | 5.90E-23 |
| ENSDARG00000044365 | angiopoietin-like 3(angptl3) | 1.3809 | 2.48E-08 |
| ENSDARG00000075159 | antigen p97 (melanoma associated) identified by monoclonal antibodies 133.2 and 96.5(mfi2) | 1.422 | 6.97E-33 |
| ENSDARG00000012076 | apolipoprotein A-Ia(apoa1a) | -1.1431 | 0 |
| ENSDARG00000042780 | apolipoprotein Ba(apoba) | 1.5617 | 0 |
| ENSDARG00000075614 | apolipoprotein C-IV(apoc4) | 1.1629 | 7.59E-05 |
| ENSDARG00000093569 | branched chain keto acid dehydrogenase E1, beta polypeptide, like(bckdhbl) | 1.0371 | 2.19E-05 |
| ENSDARG00000040510 | carbonic anhydrase XVb(ca15b) | 1.8227 | 2.30E-08 |
| ENSDARG00000029822 | carboxyl ester lipase, tandem duplicate 2(cel.2) | 1.4632 | 4.13E-45 |
| ENSDARG00000041595 | carboxylesterase 3(ces3) | 1.0955 | 6.27E-45 |
| ENSDARG00000010146 | carboxypeptidase A2 (pancreatic)(cpa2) | 1.2342 | 1.01E-12 |
| ENSDARG00000007836 | cathepsin La(ctsla) | -1.4381 | 3.57E-28 |
| ENSDARG00000074656 | cathepsin S, ortholog2, tandem duplicate 1(ctss2.1) | 1.2946 | 1.98E-07 |
| ENSDARG00000090969 | cerebellin 18(cbln18) | -2.0858 | 3.40E-08 |
| ENSDARG00000016290 | chloride channel accessory 1(clca1) | -1.2044 | 1.27E-34 |
| ENSDARG00000024032 | coagulation factor C homolog, cochlin (Limulus polyphemus)(coch) | 4.6806 | 1.53E-08 |
| ENSDARG00000036041 | coagulation factor II (thrombin)(f2) | 1.0844 | 8.27E-49 |
| ENSDARG00000029493 | coagulation factor IXb(f9b) | 1.3345 | 9.41E-05 |
| ENSDARG00000055705 | coagulation factor V(f5) | 1.1159 | 1.08E-11 |
| ENSDARG00000074908 | collagen, type VI, alpha 1(col6a1) | 1.3646 | 2.43E-16 |
| ENSDARG00000079752 | collagen, type VI, alpha 4a(col6a4a) | 1.7037 | 5.74E-06 |
| ENSDARG00000021004 | complement component 5(c5) | 1.2162 | 1.74E-32 |
| ENSDARG00000057113 | complement component 6(c6) | 1.0766 | 3.90E-06 |
| ENSDARG00000057121 | complement component 7b(c7b) | 4.1426 | 1.35E-05 |
| ENSDARG00000093068 | complement component c3b, tandem duplicate 1(c3b.1) | 1.4833 | 5.03E-49 |
| ENSDARG00000001818 | complement component c3b, tandem duplicate 2(c3b.2) | 1.2874 | 2.13E-30 |
| ENSDARG00000068290 | cytochrome P450 CYP2X12(cyp2x12) | -1.3474 | 5.61E-07 |
| ENSDARG00000068181 | dipeptidase 1 (renal)(dpep1) | -1.0817 | 8.21E-09 |
| ENSDARG00000069017 | elastin b(elnb) | 2.4911 | 6.83E-06 |
| ENSDARG00000036993 | erb-b2 receptor tyrosine kinase 3b(erbb3b) | -1.0212 | 2.15E-08 |
| ENSDARG00000006526 | fibronectin 1b(fn1b) | 1.2373 | 3.92E-245 |
| ENSDARG00000043342 | glutathione peroxidase 3(gpx3) | 1.5048 | 4.39E-18 |
| ENSDARG00000062688 | glycoprotein (transmembrane) nmb(gpnmb) | 2.1518 | 0.00015388 |
| ENSDARG00000089310 | group-specific component (vitamin D binding protein)(gc) | 1.3482 | 2.61E-28 |
| ENSDARG00000014320 | guanylate cyclase 2C(gucy2c) | -1.0638 | 7.04E-08 |
| ENSDARG00000012609 | hemopexin(hpx) | 1.6718 | 4.42E-211 |
| ENSDARG00000057498 | hyaluronan binding protein 2(habp2) | 1.1067 | 1.52E-14 |
| ENSDARG00000075864 | immunoglobulin superfamily, member 9a(igsf9a) | -1.1926 | 9.53E-08 |
| ENSDARG00000052470 | insulin-like growth factor binding protein 2a(igfbp2a) | 1.1657 | 1.20E-09 |
| ENSDARG00000031422 | insulin-like growth factor binding protein 2b(igfbp2b) | 1.4607 | 8.00E-07 |
| ENSDARG00000037836 | insulin-like growth factor binding protein, acid labile subunit(igfals) | 1.1832 | 5.73E-06 |
| ENSDARG00000007534 | intelectin 1(itln1) | 2.0842 | 3.35E-05 |
| ENSDARG00000036084 | intelectin 2(intl2) | -2.578 | 1.95E-14 |
| ENSDARG00000003523 | intelectin 3(itln3) | -1.6326 | 4.66E-07 |
| ENSDARG00000045516 | inter-alpha-trypsin inhibitor heavy chain 2(itih2) | 1.1221 | 6.77E-41 |
| ENSDARG00000055036 | inter-alpha-trypsin inhibitor heavy chain 3a(itih3a) | 1.2382 | 3.02E-15 |
| ENSDARG00000054978 | kinesin family member C3(kifc3) | -1.4035 | 3.72E-25 |
| ENSDARG00000092404 | lactase(lct) | 2.0152 | 6.23E-12 |
| ENSDARG00000086483 | leukocyte cell-derived chemotaxin-2(LOC567149) | -5.2684 | 8.56E-10 |
| ENSDARG00000053476 | lipase, hepatic a(lipca) | 1.1685 | 5.02E-09 |
| ENSDARG00000087697 | lipoprotein lipase(lpl) | 1.1256 | 0.00010028 |
| ENSDARG00000023479 | low-density lipoprotein receptor related-protein 13(lrp13) | 1.476 | 2.99E-08 |
| ENSDARG00000007988 | mannan-binding lectin serine peptidase 2(masp2) | 1.0222 | 1.71E-05 |
| ENSDARG00000017676 | matrix metallopeptidase 2(mmp2) | 1.1365 | 2.16E-05 |
| ENSDARG00000090557 | microfibril-associated glycoprotein 4-like(LOC100007488) | -1.6813 | 5.49E-05 |
| ENSDARG00000079647 | mucin 13b, cell surface associated(muc13b) | -1.0689 | 7.99E-45 |
| ENSDARG00000019521 | myeloid-specific peroxidase(mpx) | -4.1626 | 1.18E-69 |
| ENSDARG00000017441 | myosin, light polypeptide 3, skeletal muscle(mylz3) | -1.1171 | 2.83E-10 |
| ENSDARG00000068710 | nidogen 1a(nid1a) | 1.1484 | 2.42E-20 |
| ENSDARG00000095281 | nuclear receptor coactivator 6-like(LOC100537597) | 1.7486 | 1.87E-37 |
| ENSDARG00000037789 | parvalbumin 1(pvalb1) | -1.6349 | 5.97E-21 |
| ENSDARG00000031228 | podocalyxin-like(podxl) | 1.1026 | 1.63E-08 |
| ENSDARG00000090996 | prostate androgen-regulated mucin-like protein 1(parm1) | -1.0604 | 2.47E-05 |
| ENSDARG00000028163 | proteoglycan 4b(prg4b) | 1.0196 | 9.87E-06 |
| ENSDARG00000034989 | retinol saturase (all-trans-retinol 13,14-reductase) like(retsatl) | 1.731 | 7.52E-17 |
| ENSDARG00000095590 | secreted phosphoprotein 2(spp2) | 1.6735 | 4.03E-06 |
| ENSDARG00000079727 | selenoprotein P, plasma, 1b(sepp1b) | 1.0273 | 1.73E-08 |
| ENSDARG00000087143 | serpin peptidase inhibitor, clade A (alpha-1 antiproteinase, antitrypsin), member 7(serpina7) | 1.3963 | 1.97E-10 |
| ENSDARG00000042684 | serpin peptidase inhibitor, clade C (antithrombin), member 1(serpinc1) | 1.0397 | 2.32E-22 |
| ENSDARG00000021208 | serpin peptidase inhibitor, clade D (heparin cofactor), member 1(serpind1) | 1.0632 | 6.34E-10 |
| ENSDARG00000076448 | serpin peptidase inhibitor, clade F (alpha-2 antiplasmin, pigment epithelium derived factor), member 2a(serpinf2a) | 1.1449 | 2.23E-13 |
| ENSDARG00000058053 | serpin peptidase inhibitor, clade G (C1 inhibitor), member 1(serping1) | 4.62E-14 | 4.53E-12 |
| ENSDARG00000019492 | sex hormone-binding globulin(shbg) | 1.0239 | 3.15E-13 |
| ENSDARG00000090870 | si:ch1073-263o8.2(si:ch1073-263o8.2) | 1.8183 | 3.96E-22 |
| ENSDARG00000006901 | si:ch1073-459j12.1(si:ch1073-459j12.1) | 1.4715 | 6.11E-06 |
| ENSDARG00000088923 | si:ch211-12h2.8(si:ch211-12h2.8) | -1.9385 | 1.40E-22 |
| ENSDARG00000093216 | si:ch211-146l10.7(si:ch211-146l10.7) | -1.8348 | 2.71E-137 |
| ENSDARG00000045423 | si:ch211-146l10.8(si:ch211-146l10.8) | -2.0252 | 6.79E-108 |
| ENSDARG00000076534 | si:ch211-14a17.10(si:ch211-14a17.10) | 2.4066 | 5.38E-15 |
| ENSDARG00000077960 | si:ch211-186e20.7(si:ch211-186e20.7) | 1.2018 | 2.26E-06 |
| ENSDARG00000086221 | si:ch211-226h8.4(si:ch211-226h8.4) | -2.0192 | 4.09E-27 |
| ENSDARG00000056498 | si:ch211-234p6.10(si:ch211-234p6.10) | -2.5104 | 1.48E-06 |
| ENSDARG00000088251 | si:ch211-24o10.6(si:ch211-24o10.6) | 2.63 | 8.12E-22 |
| ENSDARG00000070960 | si:ch211-288g17.4(si:ch211-288g17.4) | 2.63 | 8.12E-22 |
| ENSDARG00000087508 | si:ch211-39i22.1(si:ch211-39i22.1) | -1.422 | 2.21E-14 |
| ENSDARG00000097446 | si:ch211-66i15.5(si:ch211-66i15.5) | -4.2695 | 2.21E-10 |
| ENSDARG00000087392 | si:ch73-160p18.3(si:ch73-160p18.3) | -1.8665 | 1.28E-53 |
| ENSDARG00000086247 | si:ch73-189n23.1(si:ch73-189n23.1) | -1.99 | 9.33E-25 |
| ENSDARG00000041685 | si:dkey-105h12.2(si:dkey-105h12.2) | 1.3958 | 3.41E-38 |
| ENSDARG00000079034 | si:dkey-19b23.11(si:dkey-19b23.11) | -1.8981 | 6.33E-131 |
| ENSDARG00000094392 | si:dkey-229d11.5(si:dkey-229d11.5) | -2.0945 | 1.92E-60 |
| ENSDARG00000092044 | si:dkey-22f5.9(si:dkey-22f5.9) | 1.1862 | 1.06E-08 |
| ENSDARG00000088989 | si:dkey-241l7.5(si:dkey-241l7.5) | -1.002 | 9.26E-13 |
| ENSDARG00000077171 | si:dkey-46g23.2(si:dkey-46g23.2) | -1.7732 | 2.48E-118 |
| ENSDARG00000008835 | si:dkey-46g23.5(si:dkey-46g23.5) | -2.119 | 9.15E-77 |
| ENSDARG00000095462 | si:dkey-57c15.4(si:dkey-57c15.4) | 1.1997 | 5.52E-08 |
| ENSDARG00000029388 | si:dkey-90m5.4(si:dkey-90m5.4) | 1.2582 | 7.35E-07 |
| ENSDARG00000092498 | si:dkeyp-46h3.2(si:dkeyp-46h3.2) | -1.928 | 5.93E-29 |
| ENSDARG00000092887 | si:dkeyp-46h3.3(si:dkeyp-46h3.3) | -1.7821 | 2.70E-20 |
| ENSDARG00000069251 | si:dkeyp-50f7.2(si:dkeyp-50f7.2) | -1.8411 | 7.23E-147 |
| ENSDARG00000079589 | si:dkeyp-73d8.6(si:dkeyp-73d8.6) | 1.0081 | 1.16E-12 |
| ENSDARG00000079043 | si:dkeyp-75b4.10(si:dkeyp-75b4.10) | 1.8276 | 4.69E-09 |
| ENSDARG00000070964 | si:rp71-15k1.1(si:rp71-15k1.1) | 1.2363 | 5.98E-05 |
| ENSDARG00000089966 | si:zfos-1505d6.3(si:zfos-1505d6.3) | -1.6125 | 2.98E-34 |
| ENSDARG00000026335 | sushi domain containing 4(susd4) | -1.2468 | 5.85E-06 |
| ENSDARG00000037191 | transthyretin (prealbumin, amyloidosis type I)(ttr) | 1.0052 | 2.16E-07 |
| ENSDARG00000040815 | tsukushi small leucine rich proteoglycan homolog (Xenopus laevis)(tsku) | 1.3009 | 1.03E-06 |
| ENSDARG00000045299 | vitelline membrane outer layer 1 homolog b(vmo1b) | 1.0782 | 1.79E-08 |
| ENSDARG00000055388 | vitronectin a(vtna) | 1.1386 | 5.70E-11 |
| ENSDARG00000070709 | wu:fi42e03(wu:fi42e03) | -1.8523 | 3.22E-73 |
| ENSDARG00000043175 | zgc:112368(zgc:112368) | 1.0457 | 1.01E-05 |
| ENSDARG00000008948 | zgc:123275(zgc:123275) | -2.3401 | 1.74E-05 |
| ENSDARG00000068465 | zgc:136254(zgc:136254) | -1.5157 | 8.00E-20 |
| ENSDARG00000063518 | zgc:153913(zgc:153913) | 1.2365 | 2.57E-05 |
| ENSDARG00000091558 | zgc:158494(zgc:158494) | 1.5144 | 7.33E-09 |
| ENSDARG00000069375 | zgc:162608(zgc:162608) | 1.0643 | 2.19E-06 |
| ENSDARG00000075737 | zgc:165518(zgc:165518) | 1.9482 | 1.11E-27 |
| ENSDARG00000076771 | zgc:171445(zgc:171445) | -1.8331 | 2.52E-64 |
| ENSDARG00000090822 | zgc:171517(zgc:171517) | -1.9172 | 1.35E-19 |
| ENSDARG00000016908 | zgc:171779(zgc:171779) | -1.9332 | 4.85E-66 |
| ENSDARG00000092885 | zgc:171977(zgc:171977) | -2.1048 | 4.59E-119 |
| ENSDARG00000090768 | zgc:173556(zgc:173556) | -1.8158 | 2.80E-173 |
| ENSDARG00000043168 | zgc:92511(zgc:92511) | 1.0099 | 2.64E-05 |
| ENSDARG00000055415 | zona pellucida glycoprotein 2, like 1(zp2l1) | -1.9694 | 1.51E-29 |
| ENSDARG00000086352 | zona pellucida glycoprotein 2, tandem duplicate 1(zp2.1) | -1.7205 | 3.22E-22 |
| ENSDARG00000090237 | zona pellucida glycoprotein 2, tandem duplicate 3(zp2.3) | -2.1825 | 2.03E-70 |
| ENSDARG00000086522 | zona pellucida glycoprotein 2, tandem duplicate 5(zp2.5) | -1.7684 | 4.31E-30 |
| ENSDARG00000091409 | zona pellucida glycoprotein 2, tandem duplicate 6(zp2.6) | -1.8023 | 1.31E-50 |
| ENSDARG00000042129 | zona pellucida glycoprotein 3a, tandem duplicate 1(zp3a.1) | -1.9098 | 1.41E-74 |
| ENSDARG00000042130 | zona pellucida glycoprotein 3a, tandem duplicate 2(zp3a.2) | -1.8243 | 1.42E-59 |
| ENSDARG00000039828 | zona pellucida glycoprotein 3b(zp3b) | -1.9027 | 3.69E-93 |
| ENSDARG00000059252 | zona pellucida protein C(zpcx) | -2.0339 | 1.75E-40 |
