# Supplementary material for: Impact of Lactobacillus casei BL23 on the Host Transcriptome, Growth and Disease Resistance in Larval Zebrafish
Source: Front Physiol. 2018 Sep 4;9:1245. doi: 10.3389/fphys.2018.01245 (PMC6131626; doi:10.3389/fphys.2018.01245)
Supplement: TABLE S6 — Genes involved myogenesis, growth regulation and PPAR signal pathway in zebrafish at 35 dpf, L. casei BL23 vs. control. [file Table_6.DOCX]

Table S6 Genes involved myogenesis, growth regulation and PPAR signal pathway in zebrafish at 35 dpf, *L. casei* BL23 vs control.

| Gene | Gene_id | mRNA-seq | | RT-qPCR | |
| --- | --- | --- | --- | --- | --- |
|  |  | Fold change | p-value | Fold change | p-value |
| igf1a | ENSDARG00000094132 | 3.91900714 | 0.0041506 | 4.53 | < 0.05 |
| igf2b | ENSDARG00000033307 | 1.66763039 | 0.041991 | -- | -- |
| ppar-α | ENSDARG00000054323 | 1.89234232 | 0.050545 | 4.18 | < 0.05 |
| ppar-β | ENSDARG00000044525 | 1.82260654 | 0.0098807 | 3.55 | < 0.05 |
| RAR-γ | ENSDARG00000034117 | 2.06867421 | 0.04155 | 5.05 | < 0.05 |
| igfbp2a | ENSDARG00000052470 | 2.24334273 | 1.2045E-09 | -- | -- |
| igfbp2b | ENSDARG00000031422 | 2.75249791 | 7.9959E-07 | -- | -- |
